# Incidence and risk factors for trocar-site incisional hernia detected by clinical and ultrasound examination: a prospective observational study

**DOI:** 10.1186/s12893-020-01000-6

**Published:** 2020-12-14

**Authors:** Ana Ciscar, Josep M. Badia, Francesc Novell, Santiago Bolívar, Esther Mans

**Affiliations:** 1 Department of Surgery, Hospital de Mataró, Carretera de Cirera, 230, 08304 Mataró, Barcelona Spain; 2 Department of Surgery, Hospital General de Granollers, Granollers, Barcelona Spain; 3 Universitat Internacional de Catalunya, Barcelona, Spain; 4 Department of Radiology, Hospital Parc Taulí de Sabadell, Sabadell, Barcelona Spain; 5 Department of Radiology, Hospital Universitari de Bellvitge, L’Hospitalet de Llobregat, Barcelona, Spain; 6 Universitat de Barcelona, Barcelona, Spain

**Keywords:** Trocar site incisional hernia, Laparoscopic surgery, Risk factors, Incidence

## Abstract

**Background:**

Trocar site incisional hernia (TSIH) is the most frequent complication associated with laparoscopic surgery. Few studies currently describe its incidence or risk factors. The aim of this report is to determine the real incidence of TSIH and to identify risk factors.

**Methods:**

A cross-sectional prospective study was performed including consecutive patients who underwent a laparoscopic procedure during a 4 months period*.* All the patients were assessed both clinically (TSIHc) and by an ultrasonographic examination (TSIHu). The main variable studied was the incidence of TSIH. A multivariate analysis was performed to identify risk factors.

**Results:**

76 patients were included. 27.6% of patients were clinically diagnosed as having TSIH (TSIHc) but only 23.7% of those cases were radiologically confirmed (TSIHu). In the logistic regression analysis, age > 70 years (OR 3.462 CI 1.14–10.515, p = 0.028) and body mass index (BMI) ≥ 30 kg/m^2^ (OR 3.313 CI 1.037–10.588, p = 0.043) were identified as risk factors for TSIH. The size of the trocar also showed statistically significant differences (p < 0.001). Mean follow-up time was 34 months.

**Conclusions:**

TSIH is under-diagnosed due to the lack of related symptomatology and the inadequacy of the postoperative follow-up period. We detected discrepancies between the clinical and ultrasonographic examinations. TSIHu should be considered as the gold standard for the diagnosis of TSIH. Risk factors such as age, BMI and size of the trocar were confirmed. Patients should be followed-up for a minimum of 2 years.

*Trial registration* The study has been retrospectively registered in Clinicaltrials.gov on June 4, 2020 under registration number: NCT04410744

## Background

Incisional hernia is the most common complication associated with surgical procedures, with an estimated incidence of 0–35% [1, 2]. As there are few studies with a long-term follow up and the occurrence of the incisional hernia depends on the duration of postoperative follow-up, this estimate may not be accurate.

There is a considerable amount of documentation regarding incisional hernia after laparotomy, and several risk and protective factors have been described. Despite the introduction of laparoscopic surgery, incisional hernia is still frequent and, indeed, trocar site incisional hernia (TSIH) is a common complication after laparoscopy. Until recently, even in studies with a long term follow-up, unrealistic TSIH rates of 0.8–2.9% [3–6] have been described.

TSIH can be diagnosed by clinical examination, but imaging tests as computerized tomography (CT) or dynamic abdominal sonography for hernia (DASH) can improve the diagnostic accuracy. At present, a CT scan is considered the gold standard technique for the diagnosis and characterization of TSIH, but it has a few drawbacks: economic cost, patient irradiation, and that it is a static procedure (which can under-diagnose). On the other hand, dynamic abdominal sonography for hernia (DASH) has been shown to be a valid alternative to CT in the diagnosis and characterization of incisional herniation [7, 8].

One of the key factors for proper detection of the TSIH is the postoperative follow-up time. A short or incomplete follow up could underdiagnose this issue, which is often subclinical [7].

The aim of this study was to determine the incidence of trocar-site incisional hernia (TSIH) by clinical and dynamic ultrasonographic examination in patients who underwent laparoscopic surgeries in a general hospital. Secondary outcomes were to evaluate the correlation between clinical and ultrasonographic assessment and to determine the main risk factors for TSIH.

## Methods

### Study design

A single-centre cross-sectional study based on prospective clinical and radiological assessment and retrospective risk factor analyses performed at a single hospital. The study has been retrospectively registered in Clinicaltrials.gov on June 4, 2020 under registration number: NCT04410744.

### Patients

All consecutive patients undergoing laparoscopic surgery (cholecystectomy, colon resection, adrenalectomy, Nissen fundoplication and appendectomy) during a 4-month period were included in the study. 30 months after hospital discharge, they were invited by telephone to participate in the study. Detailed project information was provided, and those who accepted received an appointment for an outpatient visit and a dynamic ultrasound examination. A written informed consent was obtained from all participants. Exclusion criteria were age under 18 years, previous umbilical hernia repair, or failure to attend the postoperative appointment.

### Procedures

In all patients, the umbilical trocar wound was closed with an interrupted suture with synthetic braided absorbable 2/0 suture (Novosyn^®^ or Safil^®^). During the postoperative outpatient visit, informed consent was obtained and clinical and ultrasonographic examinations were performed. To increase study homogeneity, a single surgeon performed all clinical examinations and all ultrasound examinations were performed by a radiology medical trainee closely supervised by the same abdominal radiology consultant. Sonography was performed using an Applio 500 equipment (Toshiba, Japan) with a 7 MHz linear probe. The ultrasound diagnosis was based on the identification of an abdominal wall defect with intraabdominal tissue protrusion. Caudal and cranial diameters of the abdominal wall’s defect were recorded. Patients diagnosed with TSIH were offered elective repair.

### Measurements and variables

The main variables of the study were clinical (TSIHc) and ultrasound (TSIHu) diagnosis of incisional hernia. Secondary variables analysed were age, sex, diabetes mellitus, chronic obstructive pulmonary disease (COPD), smoking, body mass index (BMI), previous untreated umbilical hernia, malignancy, surgical time, urgent/elective indication, incision size, degree of contamination and surgeon’s experience.

### Statistical analysis

All results and variables were entered into a specifically designed database (File MakerPro 11.0v3 © 1984–2011 FileMaker, Inc.). Data were collected from clinical interviews, physical and radiological examinations and the electronic medical record. Continuous variables were described as means and standard deviations and categorical variables were described as absolute numbers and percentages. The Chi-square test was used to compare categorical variables (Fisher’s exact test was used when needed), and the Student t-test was used to compare continuous variables. Bivariate analysis and multivariate logistic regression analysis were performed to identify independent predictive causal factors for the development of TSIH. Adjusted odds ratios (ORs) were calculated using logistic regression. Variables achieving statistical significance in the bivariate analysis were considered for multivariate analysis. ORs with 95% confidence intervals (CIs) were presented for each studied variable. Differences were significant at the 5% level. All reported *p* values were two-sided. Statistical analyses were performed using SPSS statistical software (IBM SPSS^®^ Statistics).

## Results

**Fig. 1.**
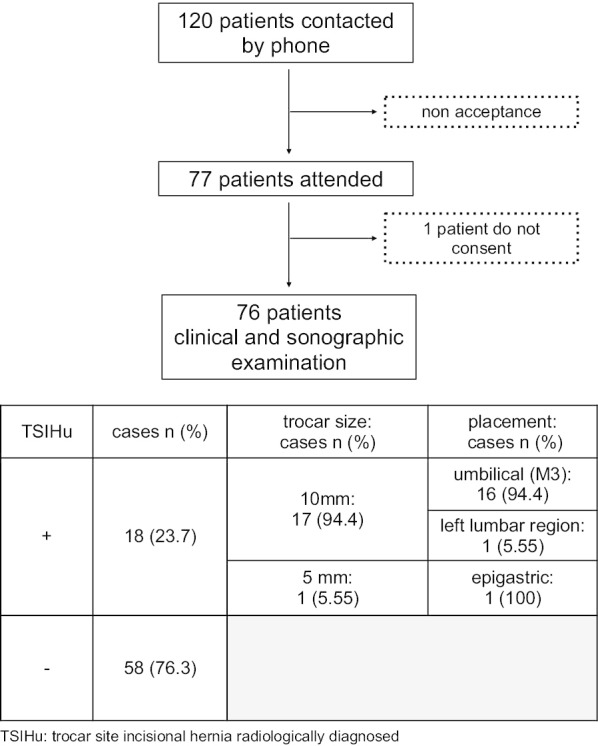
Chart

A total of 120 patients, who were operated on, were contacted and 76 of them were finally included in the study (Fig. 1). 57.9% were women, and the mean age was 58.5 years. Among them, 13.2% were diabetic, 5.4% had chronic obstructive pulmonary disease (COPD), 27.5% were obese (BMI over 30 kg/m^2^) and 11.8% had a malignancy. The primary laparoscopic procedures were cholecystectomy (78.9%), sigmoidectomy (10.5%), Nissen fundoplication (7.9%), adrenalectomy (1.3%) and appendectomy (1.3%). Mean postoperative follow-up was 34 months (Table 1).

**Table 1:** Main patient characteristics

N	76
Age years (SD)	58.47 (16.58)
≤ 70 n (%)	54 (71.1)
> 70	22 (28.9)
Gender n (%)	
Male	32 (41.1)
Female	44 (57.9)
DM n (%)	
Yes	10 (13.2)
Not	66 (86.8)
COPD n (%)	
Yes	4 (5.4)
Not	70 (94.6)
Smokers n (%)	
Yes	19 (27.1)
Not	51 (72.9)
BMI kg/m^2^ (SD)	27.54 (4.25)
≥ 30 n (%)	19 (27.5)
< 30	50 (72.5)
Weight kg, (SD)	74.51 (11.76)
Height cm (SD)	163.82 (8.31)
Previous umbilical hernia n (%)	
Yes	7 (9.3)
Not	68 (90.7)
Surgery type n (%)	
Cholecystectomy	60 (78.9)
Sigmoidectomy	8 (10.5)
Nissen fundoplication	6 (7.9)
Suprarrenalectomy	1 (1.3)
Appendectomy	1 (1.3)
Laparoscopic approach n (%)	
Hasson	76 (100)
Veress	0
Operative time minutes (SD)	86.89 (44.13)
Surgeon’s experience n (%)	
Junior	9 (12)
Senior	66 (88)
Diagnose n (%)	
Cholelithiasis	39 (51.3)
Acute cholecystitis	16 (21.1)
Colon cancer	9 (11.8)
Hiatal hernia	5 (6.6)
Chronic cholecystitis	4 (5.3)
Porcelain Gallbladder	1 (1.3)
Adrenal adenoma	1 (1.3)
Acute appendicitis	1 (1.3)
Malignancy n (%)	
Benign	67 (88.2)
Malign	9 (11.8)
Surgical wound infection classification n (%)	
Clean	8 (10.5)
Clean–contaminated	44 (57.9)
Contaminated	24 (31.6)
Emergency n (%)	
Elective	51 (67.1)
Urgent	25 (32.9)
TSIHc n (%)	21 (27.6)
5 mm/TSIH	1 (4.76)
10 mm/TSIH	20 (95.23)
TSIHu n (%)	18 (23.7)
5 mm/HLA TSIH	1 (5.5)
10 mm/HLA TSIH	17 (94.45)
Symptomatic	8 (47.1)
Asymptomatic	10 (52.9)
Content n (%)	
Omentum	13 (70.6)
Small bowel	3 (17.6)
Both	2 (11.8)
Transverse diameter mm (SD)	13.39 (7.55)
Cranio caudal diameter mm (SD)	12.37 (8.52)
Follow up months (SD)	33.66 (4.36)

*DM* diabetes mellitus, *COPD* chronic obstructive pulmonary disease, *BMI* body mass index, *TSIHc* trocar site incision hernia clinically diagnosed, *TSIHu* trocar site incision hernia diagnosed radiologically

### TSIH incidence

Of the 76 patients included in the study, a total of 303 trocar site incisions (TSI) were assessed (151 of 10 mm-TSI and 152 of 5 mm-TSI). A total of 21 patients (27.6%) were clinically diagnosed with having TSIH (TSIHc). However, TSIH was confirmed radiologically (TSIHu) in only 18 cases (23.7%). No patient presented more than one TSIH. Only 47.1% of patients who had a confirmed TSIH presented symptoms.

Differences were observed in the TSIH rate depending on the size and location of the trocar. Only 1 out of 18 (5.5%) TSIH occurred in a 5-mm trocar site, whereas the other 17 hernias (94.45%) were located in 10-mm trocar sites. All hernias were located in the umbilical area (M3), except for one located in the left lumbar region (L4).

Nine cases of TSIH (15%) suspected by clinical examination were not confirmed following ultrasonography. On the contrary, five cases (28%) diagnosed by ultrasonographic examination were not clinically found. Ultrasonography showed a significant better diagnostic capacity than clinical examination (p < 0.001).

The five not clinically suspected cases showed a lower transverse and cranio-caudal diameter measurement (Table 2), although there were not statistical differences.

**Table 2:** TSIHu positive cases

	TSIHc+	TSIHc−	p
CC diameter (mm, SD)	13.5 (9.8)	8.6 (3.8)	0.16
T diameter (mm, SD)	14.17 (8.8)	11.2 (4.3)	0.372
BMI (kg/m^2^, SD)	30.04 (3.3)	28.26 (5.3)	0.517

*TSIHu* ultrasound trocar site incision hernia, *TSIHc* clinical trocar site incision hernia, *CC* craniocaudal, *T* transverse, *BMI* body mass index

### Risk factors for TSIH

Table 3 shows the differences observed between patients with TSIH and those with no TSIH identified radiologically. Only age > 70 and BMI ≥ 30 showed statistically significant differences. In the univariate analysis of potential risk factors for TSIH, statistically significant differences were observed for age > 70 years (OR 3.462; 95% CI 1.14–10.515; p = 0.028) and BMI ≥ 30 (OR 3.313; CI 1.037–10.588; p = 0.043) (Table 4). Other variables were discarded. Both, age > 70 years (OR 4.464; 95% CI 1.32–15.091; p = 0.016) and BMI ≥ 30 (OR 3.572; 95% CI 1.034–12.338; p = 0.044) were confirmed as risk factors by multivariate analyses (Table 5).

**Table 3:** Comparison between groups

Variable	TSIHu+	TSIHu−	p
Age years (SD)	62.56 (17)	57.21 (16.4)	
≤ 70 n (%)	9 (50)	45 (77.5)	0.019^¶^
> 70	9 (50)	13 (22.4)	0.037^§^
Gender n (%)			0.752^§^
Male	7 (39)	25 (43)	
Female	11 (61)	33 (57)	
Diabetes mellitus n (%)			1^ƒ^
Yes	2 (11)	8 (13.8)	
No	16 (88)	50 (86.2)	
COPD (%)			0.247^ƒ^
Yes	2 (11)	2 (3.6)	
No	16 (88)	54 (96.4)	
Smoker (%)			
Yes	7 (41.2)	12 (22.6)	0.135^§^
No	10 (58.8)	41 (77.4)	
BMI kg/m^2^ (SD)	29.66 (3.89)	26.89 (4.17)	
≥ 30	8 (47)	11 (21.2)	0.019^†^
< 30	9 (53)	41 (78.8)	0.038^§^
Weight kg (SD)	77.47 (10.21)	73.53 (12.16)	0.234^†^
Height cms, SD	161.29 (7.49)	164.67 (8.46)	0.149^†^
Previous umbilical hernia n (%)			
Yes	3 (1,7)	4 (7)	0.34^ƒ^
No	15 (83)	53 (93)	
Malignancy n (%)	2 (11.1)	7 (12)	1^ƒ^
Wound infection classification (%)			0.371^§^
Clean	2 (11)	6 (10.3)	
Clean–contaminated	8 (44)	36 (62.1)	
Contaminated	8 (44)	16 (27.6)	
Emergency n (%)			0.536^§^
Urgent	7 (38.9)	18 (31)	
Elective	11 (61.1)	40 (69)	
Surgeon’s experience (%)			0.441^ƒ^
Junior	3 (17)	6 (10.5)	
Senior	15 (83)	51 (89.5)	
Operative time (minutes)	94.33 (41.3)	84.6 (45)	0.417^†^
Follow up (months)	33.17 (5.31)	33.825 (4.05)	0.582^†^

^§^Chi square

^ƒ^f Fisher

^†^t Student

^¶^U de Mann–Whitney

**Table 4:** Results of univariate logistic regression analysis. Identification of risk factors

Variable	OR	95%IC	p
Age (years)			
≤ 70	Reference	1.14–10.515	0.028
> 70	3.462		
BMI (kg/m^2^)			
< 30	Reference	1.037–10.588	0.043
≥ 30	3.313

**Table 5:** Results of multivariate logistic regression analysis. Identification of risk factors

Variable	OR	95%IC	p
Age (years)			
≤ 70	Reference	1.320–15.091	0.016
> 70	4.464		
BMI (kg/m^2^)			
< 30	Reference	1.034–12.338	0.044
≥ 30	3.572		

## Discussion

Incisional hernia is defined as a defect in the abdominal wall in placements of postoperative wounds. They are recognizable by clinical examination and/or by imaging tests. The incidence of trocar site incisional hernia has been poorly documented over the years which may be due to the paucity of symptoms and to the lack of long-term postoperative follow-up since the pathology that leads to the laparoscopic surgery is usually benign and no further follow-up is deemed necessary. Consequently, in most situations the follow-up is not sufficient to detect TSIH.

In the present study it was hypothesized that the real incidence of trocar site incisional hernia, when properly assessed, could be higher than is currently believed. Therefore, we aimed to analyze the actual TSIH incidence in our environment, measured by both physical and radiological exams, the latter being considered the gold standard technique [8].

### Incidence

In our series, a high TSIH rate was found. The incidence detected by physical examination was 27.6%, compared to 23.7% when assessed by ultrasound. After having been a neglected issue, during the last decade, few authors have addressed the incidence and risk factors of TSIH after laparoscopic surgery. In 2010, Chiong et al. published a retrospective analysis of 1055 patients who underwent surgery due to urologic tumors and found a TSIH rate of 0.66%. All of them were clinically suspected and radiologically confirmed by computed tomography [9]. In 2011, a systematic review based on 19 prospective and retrospective studies, which included a total of 30,568 adults and 1098 children, documented a TSIH incidence of 0.5–2% [10]. In 2013, a retrospective review of 500 patients who underwent laparoscopic and robotic gynecological surgery documented only three cases of TSIH (0.6%), diagnosed on physical examination with radiologic confirmation (1 of them required emergency reoperation for hernia reduction and the other two presented asymptomatic bulges), with an average length of time to TSIH appearance of 21 days [4]. In 2011, in a narrative review, Comajuncosas et al. described an incidence of 0.18–2.8%, but the authors concluded that the actual incidence was possibly higher [11]. Three years later, the same group published a prospective observational study including 241 patients, with a follow-up of 46.8 months, showing an incidence of 25.9%. In this study, TSIH were identified mainly with clinical examination, but an abdominal ultrasound was carried out in doubtful cases [12].

### Location

In spite of using a systematic protocol for closure, most of TSIH cases were located in the 10 mm incisions at the umbilical level. The TSIH rate can change depending on the type of trocar and its location. TSIH have been described at any location, but those situated at the 10 mm trocar are the most frequent [9, 10, 12–14]. It seems that for 5 mm trocars facial closure should not be necessary, but for ≥ 10 mm trocars it would be mandatory. Keeping in mind some previous studies [4, 6, 10, 11] and the results if this study, the standard closure technique may not be enough.

### Risk factors

We found that obesity and age over 70 years were independent risk factors for the appearance of TSIH. In open surgery, some risk factors for incisional hernias, either individual or dependent on the surgical technique, are well documented. Factors including abdominal aortic aneurysm surgery, obesity, cachexia, advanced age, male sex, COPD, anemia, smoking, steroid treatment, and immunosuppression [1, 10, 12, 15, 16]. Duration of the procedure, presence of previous umbilical hernia, diabetes mellitus, or smoking, have been described as possible risk factors for TSIH [11, 17] but according to our data only age and obesity can be confirmed as risk factors for TSIH.

### Diagnosis

In our experience, clinical examination overdiagnoses TSIH which is quite surprising and is not in accordance with other authors. A systematic review carried out in 2018 which was led by Kroese et al. [18] concluded that the use of imaging modalities would usually result in more incisional hernia being diagnosed compared to the use of physical examination alone. Bloemen et al. [19] also concluded that performing an ultrasonographic as well as a physical examination yielded a significant number of hernias, mostly asymptomatic ones, which would not have been found using physical examination alone.

### Follow up

As in open surgery, one of the main problems when considering laparoscopic incisional hernia diagnosis is a proper period of follow-up. There is no certainty about which would be the optimal follow-up time to detect TSIH and most studies describe follow-up for less than 1 year. In addition, the frequent absence of symptoms would result in a lack of medical consulting. According to the definition of Tonouchi [17], it seems reasonable to advise a minimum follow-up of 2 years, although some authors recommend more than 4 years [12, 13]. The mean follow up in our series almost achieves 3 years (34 months) which would be quite reasonable.

The study has some limitations: there may be some selection bias, as the acceptance could be related to the presence of symptoms and cancer patients having exhaustive oncological controls may have refused to participate so as not to increase the number of outpatient consultations. The fact that the clinical evaluation was performed only by a single physician could also reduce the generalizability of our results. Finally, the relatively small sample size was enough to determine the prevalence of TSIH, but it was probably suboptimal for the calculation of risk factors.

## Conclusions

We conclude that TSIH is underdiagnosed in most of the published series. Diagnosing TSIH requires an exhaustive study that includes not only physical examination, but also ultrasonography. A significant number of patients ultimately diagnosed with TSIH hadn’t previously reported symptoms. Age, BMI and the size of the incision are factors that lead to the appearance of TSIH.

Remaining questions for further investigation are whether all patients diagnosed with TSIH must be operated on, and whether the high incisional hernia rate justify changes in operative techniques, such as improvements in trocar closure, prophylactic mesh placement or lateralization of the umbilical 10 mm trocar.

## Data Availability

The datasets used and/or analysed during the current study are available from the corresponding author on reasonable request.

## Abbreviations

TSIH: Trocar site incisional hernia
TSIHc: Trocar site incisional hernia assessed clinically
TSIHu: Trocar site incisional hernia assessed by ultrasound examination
OR: Odds ratio
CI: Confidence intervals
CT: Computerized tomography
DASH: Dynamic abdominal sonography for hernia
BMI: Body mass index
COPD: Chronic obstructive pulmonary disease
TSI: Trocar site incision
L4: Left lumbar region
M3: Umbilical region
